# Spatiotemporal multi-disease transmission dynamic measure for emerging diseases: an application to dengue and zika integrated surveillance in Thailand

**DOI:** 10.1186/s12874-019-0833-6

**Published:** 2019-10-26

**Authors:** Chawarat Rotejanaprasert, Andrew B. Lawson, Sopon Iamsirithaworn

**Affiliations:** 1 Department of Tropical Hygiene, Faculty of Tropical Medicine, Mahidol University, Ratchathewi, Bangkok, 10400 Thailand; 2 Mahidol-Oxford Tropical Medicine Research Unit, Faculty of Tropical Medicine, Mahidol University, Bangkok, 10400 Thailand; 3 Department of Public Health Sciences, Medical University of South Carolina, Charleston, SC 29425 USA; 4 Department of Disease Control, Ministry of Public Health, Nonthaburi, 11000 Thailand

**Keywords:** Emerging disease, Surveillance, Integrative, Bayesian, Spatiotemporal, Zika, Dengue

## Abstract

**Background:**

New emerging diseases are public health concerns in which policy makers have to make decisions in the presence of enormous uncertainty. This is an important challenge in terms of emergency preparation requiring the operation of effective surveillance systems. A key concept to investigate the dynamic of infectious diseases is the basic reproduction number. However it is difficult to be applicable in real situations due to the underlying theoretical assumptions.

**Methods:**

In this paper we propose a robust and flexible methodology for estimating disease strength varying in space and time using an alternative measure of disease transmission within the hierarchical modeling framework. The proposed measure is also extended to allow for incorporating knowledge from related diseases to enhance performance of surveillance system.

**Results:**

A simulation was conducted to examine robustness of the proposed methodology and the simulation results demonstrate that the proposed method allows robust estimation of the disease strength across simulation scenarios. A real data example is provided of an integrative application of Dengue and Zika surveillance in Thailand. The real data example also shows that combining both diseases in an integrated analysis essentially decreases variability of model fitting.

**Conclusions:**

The proposed methodology is robust in several simulated scenarios of spatiotemporal transmission force with computing flexibility and practical benefits. This development has potential for broad applicability as an alternative tool for integrated surveillance of emerging diseases such as Zika.

## Background

The nature of infectious diseases has been changing rapidly in conjunction with dramatic societal and environmental changes. This is a substantial challenge in terms of emergency preparedness requiring the implementation of a wide range of surveillance policies. The recent emergence of Zika outbreaks associated with birth defects prompted the World Health Organization (WHO) to declare a public health emergency of international concern in February 2016 [1]. After that, there has been an explosion in research and planning as the global health community has turned their attention to understanding and controlling Zika virus. However, the lack of important information needed to assess the global health threat from the virus still remains [2]. The behavior of an infectious disease is often formidable or sometimes not feasible to be evaluated by conducting experiments with real communities. As a result, mathematical models explaining the transmission of infectious diseases are a valuable tool for planning disease-management policies.

An important question when a new emerging disease occurs is the disease transmission mechanism and how infectious the disease is. A key concept in epidemiology to indicate the scale and speed of spread in a susceptible population is the transmissibility of the infection, characterized by the basic reproduction number, *R*_0_. This quantity has a definition of longer-term endemicity in a given population (*R*_0_ < 1 stops an epidemic) [3]. An extensive range of estimation methods have been proposed (see examples [4, 5]). Although the basic reproduction number can be useful for understanding the transmissibility of an infectious disease, the methods based on fitting deterministic transmission models are often difficult to use and generalize in practice due to context-specific assumptions which often do not hold [6, 7].

It is of practical importance to consider a computationally feasible and robust methodology to evaluate the force of infection. It has been proposed that the time course of an epidemic can be partly achieved by estimating the effective (instantaneous) reproduction number [8, 9]. However, since the contact rates among people may differ due to differences the local environment, human behavior, vector abundance, and, potentially, interactions with other viruses, disease transmissibility will vary across locations as well. Although spatial heterogeneity has been considered (see examples [10, 11]), the reproduction numbers are estimated separately for single areas without accounting for spatial variation and overdispersion in modeling. Due to very limited information when new emerging infection initially occurs, it is natural to look for strategies that mirror relevant information. Surveillance systems have been operated singly for various types of infectious diseases, however with multiple streams of geo-coded disease information available it is important to be able to take advantage of the multivariate health data. The benefits of multivariate surveillance lie in the ability to observe concurrency of patterns of disease and to allow conditioning of one disease on others. To assist public health practitioners to assess disease transmissibility in field settings, we thus develop the proposed methodology to allow for incorporating spatiotemporal knowledge from related diseases to enhance performance of surveillance system which was not considered in previous studies.

The aim of this study is to develop a generic and robust methodology for estimating spatiotemporally varying transmissibility that can be instantaneously computed for each location and time within a user-friendly environment for real-time surveillance. Not only our method has a practical interpretation with theoretical foundation but also can be understood and applied by non-technical users. The proposed method is defined in the next section with a simulation study to demonstrate robustness of the methodology. A case study is also provided of an application of integrative surveillance of Dengue and Zika virus activities in Thailand.

## Methods

### Spatiotemporal measure of disease transmission

The basic reproduction number is one of the principal concepts widely used as an epidemiological measure of the transmission potential which is theoretically defined as the number of secondary infections produced by a single infectious individuals in a susceptible population [3, 12]. However, not withstanding the issues with underlying theoretical assumptions such as population susceptibility and dynamic nature of infectious diseases, the basic reproduction number is difficult to apply in real situations. For instance, few epidemics are only ever observed when a new infection enters in a susceptible population in which the disease can also persist but had not been or able to be detected for a period of time. This situation violates a primary assumption about the ‘at risk’ population which commonly appears in new emerging diseases such as Zika. Another example is that the nature of infectious diseases is dynamic and should be consistently monitored whereas the basic reproduction number is an infinite measure which then fails to satisfy the dynamic behavior of infections. Therefore, we need to be cautious about the underlying model assumptions when applying the concept. Otherwise, it could lead to inappropriate disease-management policies or even uncontrollable outbreaks (see more discussion in [6, 7, 13]). In this paper we propose an alternative measure of spatiotemporally varying disease transmission, which we will call the surveillance reproduction number. Not affected by context-specific restrictions, this measure provides a practical interpretation that can be flexibly applied to many applications in infectious disease epidemiology. Moreover this measure which accounts for spatiotemporal heterogeneity can robustly estimate the disease strength simultaneously for all areal units and time periods, and is ideally suitable for emerging disease surveillance. To derive the proposed methodology, compartment modeling is reviewed as the foundation of our development.

There are various forms of compartmental models for infectious diseases (see examples in [5, 14]). One of the early modeling contributions is the Kermack-McKendrick model [15], a compartmental model with formulation of flow rates between disease stages of a population. A special case of the model is the well-known *SIR* (susceptible-infectious-recovered) model. A *SIR* model is usually used to describe a situation where a disease confers immunity against re-infection, to indicate that the passage of individuals is from the susceptible class *S* to the infective class *I* and to the removed class *R*. A common *SIR* model used to describe the disease at location *i* and time *t* can be specified as follows:

1 $$ {\displaystyle \begin{array}{l}\frac{dS_{it}}{dt}=-{a}_{it}{S}_{it}\\ {}\left(\frac{\partial }{\partial t}+\frac{\partial }{\partial l}\right){I}_{it}(l)={a}_{it}{S}_{it}-{b}_{it}{I}_{it}(l)\\ {}\frac{dM_{it}}{dt}={\int}_0^{\infty }{b}_{it}{I}_{it}(l) dl\end{array}} $$

where *I*_*it*_(0) = *a*_*it*_*S*_*it*_. Denote the numbers of susceptible and recovered (removed) individuals by *S*_*it*_ and *M*_*it*_. Note that we use *M* for the removed to avoid notation confusion with the surveillance reproduction number that will be constructed later. *I*_*it*_(*l*) is the number of infected individuals at time *t* with the infectious period *l*. *l* is the time elapsed since infection which is the time period of being infectious since the person got infected. *b*_*it*_(*l*) is the recovery rate during *l*. *a*_*it*_ is known as disease transmissibility at time *t* which is defined later. as ${a}_{it}={\int}_0^{\infty }{c}_{it}(l){I}_{it}(l) dl$ where *c*_*it*_(*l*) is the rate of secondary transmission per single infectious case. Although infectious modeling is usually described in a preferential sampling setting in which locations are spatially modeled, one should be aware of possible bias due to the selective sampling scheme [16, 17]. Alternatively, our methodology is developed in a conditional framework by instead conditioning the aggregated count on a fixed areal unit such as county or health district.

The disease dynamic is assumed to follow a Poisson process such that the incidence rate at which someone infected in time *t* − 1 generates new infections in time step *t* at location *i* is *μ*_*it*_. The relationship between the incidence rate *μ*_*it*_ and the prevalence *I*_*it*_ is assumed to be *I*_*it*_(*l*) = *h*_*it*_(*l*)*μ*_*it* − *l*_ for *t* − *l* > 0 where *h*_*it*_(*l*) > 0, *l* > 0, is a proportional constant, and *μ*_*it*_ = *I*_*it*_(0). The incidence rate, the number of susceptible individuals get infected, at location *i* and time *t* equals *a*_*it*_*S*_*it*_, i.e. *μ*_*it*_ = *a*_*it*_*S*_*it*_. The transmissibility ${a}_{it}={\int}_0^{\infty }{c}_{it}(l){I}_{it}(l) dl$ can be seen as the force of infection or rate at which susceptible people get infected. For example, this quantity increases if a person has a respiratory disease and does not perform good hygiene during the course of infection or decreases if that person rests in bed. Then we have that

2 $$ {\mu}_{it}={a}_{it}{S}_{it}={\int}_0^{\infty }{c}_{it}(l){S}_{it}(l){h}_{it}(l){\mu}_{it-l} dl. $$

Let *ζ*_*it*_(*l*) = *c*_*it*_(*l*)*S*_*it*_(*l*)*h*_*it*_(*l*). *ζ*_*it*_(*l*) reflects the reproductive power or effective contact rate between infectious and susceptible individuals at calendar time *t*, location *i* and infected time *l*.

To define and develop the surveillance reproduction number, *R*_*s*. *it*_, we further assume that there exist two sets of functions, ***R***_*s*, *it*_ = { *R*_*s*, *it*_ }, the set of surveillance reproduction numbers, and ${\boldsymbol{G}}_{it}=\left\{\;{g}_{it}(l)|{\int}_0^{\infty }{g}_{it}(l)\  dl=1\;\right\}$, distributional functions over the infectious time at each location, such that *ζ*_*it*_(*l*) can be decomposed into a product of those functions, i.e., *ζ*_*it*_(*l*) = *R*_*s*, *it*_*g*_*it*_(*l*). There are a number of functions in those sets satisfying the conditions. A non-trivial solution can be defined as

3 $$ {\int}_0^{\infty }{\zeta}_{it}(l)\; dl={\int}_0^{\infty }{R}_{s, it}{g}_{it}(l)\; dl={R}_{s, it}{\int}_0^{\infty }{g}_{it}(l)\; dl={R}_{s, it}. $$

However, this leads to the same issue as the basic reproductive number that we usually do not know the number of susceptible people for a given location and time which would not be very useful in field settings of emerging diseases. Hence we define the surveillance reproduction number in which ${\mu}_{it}={\int}_0^{\infty }{\zeta}_{it}(l){\mu}_{it-l} dl$. That is

4 $$ {\mu}_{it}={\int}_0^{\infty }{R}_{s, it}{g}_{it}(l){\mu}_{it-l} dl={R}_{s, it}{\int}_0^{\infty }{g}_{it}(l){\mu}_{it-l} dl $$

and, therefore, we have that

5 $$ {R}_{s, it}=\frac{\mu_{it}}{\int_0^{\infty }{g}_{it}(l){\mu}_{it-l} dl}. $$

Since ${\int}_0^{\infty }{g}_{it}(l)\  dl=1$, *R*_*s*, *it*_ can also be interpreted as the ratio of the current incidence rate to the total (weighted sum) infectiousness of infected individuals. Because patient’s information is often collected in a discrete fashion, then *R*_*s*, *it*_ can be estimated as ${R}_{s, it}\approx \frac{\mu_{it}}{\sum_{l=1}^L{g}_{it}(l){\mu}_{it-l}}$ where *L* is the maximum period of infection. Thus this quantity represents force of infection as the number of secondary infected cases that each infected individual would infect averaged over their infectious lifespan in at location *i* during time *t.* However, it is hard to derive incidence density rates due to the lack of monitoring of individual new cases and real exposed population required during a given time period and location. Then we assume that ${\mu}_{it}={h}_{it}^{\hbox{'}}{I}_{it}$ where ${h}_{it}^{\hbox{'}}>0$ is a proportional constant between prevalence and incidence at calendar time *t* and location *i*. Then the surveillance reproduction number can be expressed as

6 $$ {R}_{s, it}=\frac{h_{it}^{\hbox{'}}{I}_{it}}{\int_0^{\infty }{g}_{it}(l){h}_{it-l}^{\hbox{'}}{I}_{it-l} dl}\approx \frac{I_{it}}{\int_0^{\infty }{g}_{it}(l){I}_{it-l} dl}. $$

Hence, to estimate the surveillance number with prevalence, the ratio of incidence and prevalence is assumed to be nearly constant over time. This is a limitation of our development. This assumption may not be appropriate for long duration diseases such as chronic conditions but rather suitable for infections with relatively short duration.

The proposed methodology has practical advantages over the traditional basic reproduction number. One of which is that our method is based on prevalence, not affected by the assumption about susceptibility which is often difficult or infeasible to know. Another, since our metric is dynamic (does not depend on the infinite definition), it can be sequentially calculated which is very suitable for monitoring the disease strength, ideally for emerging diseases.

To account for spatiotemporal variation and overdispersion, *μ*_*it*_ is modeled to link to a linear predictor consisting of local variables such as environmental and demographic factors and space-time random effects to account for spatiotemporal heterogeneity as log(*μ*_*it*_) = *α* + ***X***_*it*_***β***_*it*_ + *u*_*i*_ + *v*_*i*_ + *λ*_*t*_ + *δ*_*it*_. The correlated (*u*_*i*_) and uncorrelated (*v*_*i*_) spatial components have an intrinsic conditional autoregressive (ICAR) prior distribution and zero mean Gaussian distribution respectively. In addition, there are a separate temporal random effect (*λ*_*t*_) and a space-time interaction term (*δ*_*it*_) in the linear predictor. Often the temporal effect is described using an autoregressive prior distribution such allowing for a type of nonparametric temporal effect. Note that this distribution is a random walk prior distribution with one-unit lag. For the interaction term, the prior structure is usually assumed to be distributed as a zero mean Gaussian distribution. The estimate of the reproduction number is however also dependent on the choice of the infectiousness profile, *g*_*it*_(*l*), which assumes to be Log-Normal distributed and standardized to sum to one.

Let *y*_*it*_ be the number of new cases at location *i* time *t* and the disease transmission is presumably modeled with a Poisson process. However, the cases are usually reported at a discrete time such as weekly or monthly. Assuming the transmissibility remains in the time interval (*t*, *t* + 1], the incidence at location *i* time *t* is Poisson distributed with mean *μ*_*it*_. Then the full model specification is as follows:

7 $$ {\displaystyle \begin{array}{l}{y}_{it}\sim Poisson\left({\mu}_{it}\right)\\ {}\log \left({\mu}_{it}\right)=\alpha +{\boldsymbol{X}}_{it}{\boldsymbol{\beta}}_{it}+{u}_i+{v}_i+{\lambda}_t+{\delta}_{it}\kern1em \\ {}\alpha \sim N\left(0,{\tau}_{\alpha}^{-1}\right);{\beta}_{it}\sim N\left(0,{\tau}_{\beta}^{-1}\right)\\ {}{u}_i\sim ICAR\left({\tau}_u^{-1}\right);{v}_i\sim N\left(0,{\tau}_v^{-1}\right)\\ {}{\lambda}_t\sim N\left({\lambda}_{t-1},{\tau}_{\lambda}^{-1}\right)\\ {}{\delta}_{it}\sim N\left(0,{\tau}_{\delta}^{-1}\right)\end{array}}\kern0.5em {\displaystyle \begin{array}{l}{R}_{s, it}=\frac{\mu_{it}}{\sum_{l=1}^L{g}_{il}{\mu}_{it-l}}\\ {}{g}_{il}=\frac{\exp \left({w}_{il}\right)}{\sum_{l=1}^L\exp \left({w}_{il}\right)}\\ {}{w}_{il}\sim N\left(0,{\tau}_w^{-1}\right)\\ {}{\tau}_{\ast}^{-1/2}\sim Unif\left(0,10\right).\end{array}} $$

### Simulation study

**Fig. 1:**
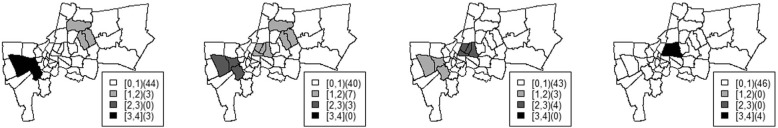
Bangkok maps of simulated *R*_*s*_ during weeks 5, 10, 15, and 20 (left-right)

**Fig. 2:**
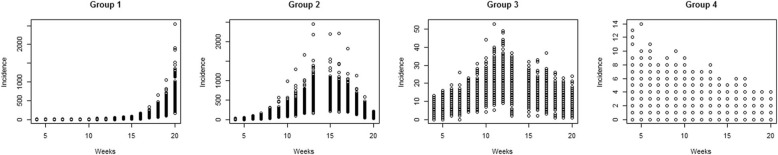
Simulated incidence of districts in group 1 (increasing *R*_*s*_), group 2 (decreasing *R*_*s*_), group 3 (with a jump) and groups 4 (constant *R*_*s*_ = 0.8)

To evaluate our proposed methodology, we simulate data without covariates in several situations with different magnitudes of transmissibility. The simulation map used as a basis for our evaluation is the district map of the province of Bangkok, Thailand. This province has 50 districts (*i* = 1–50) with a reasonably regular spatial distribution. The simulated incidence are generated for 20 weeks (*t* = 1–20) in four district groups with different levels of the reproduction numbers. Figure 1 displays the maps showing locations of simulated *R*_*s*_ of each district group at weeks 5, 10, 15, and 20. The simulated incidence of each district group with different levels of *R*_*s*_ is shown in Fig. 2 in which each dot represents a simulated value from a given simulation set. The first group (middle region in Fig. 1) is simulated with increasing magnitudes of transmission as *R*_*s*, *it*_ = 0.2 + (*t* × 0.15). The *R*_*s*, *it*_ is assumed to be increasing every time period by the size of 0.15. Then incidence with an exponentially increase is generated in this scenario to represent regions with an outbreak (left panel in Fig. 2). The second district group (western region in Fig. 1) is assumed to have decreasing magnitudes simulated as *R*_*s*, *it*_ = 4.0 − (*t* × 0.2). As can be seen in Fig. 2 (second panel from the left), the incidence in this scenario increases at the beginning due to strongly positive force of infection but will be decreasing afterwards. In the third scenario (eastern region in Fig. 1), *R*_*s*, *it*_ is assumed to have the size of 1.5 until week 12 and then reduced to 0.8 afterwards. This scenario represents the situation where an effective intervention is introduced to control an outbreak. The rest of the districts are assumed to have a constant low infection rate at *R*_*s*, *it*_ = 0.8 over the 20 time periods. To sample the discrete weight *w*_*il*_ is drawn from a normal distribution with mean of 1.5 with standard deviation of one. The infectious time, *L*, of 3 weeks is set to generate the incidence.

We generate 100 simulated incidence datasets starting with the number of newly infected people as 2, 1, and 6 for the first 3 weeks. For weeks *t* > 3, the new cases *y*_*it*_ are sampled from a Poisson distribution for each location with mean ${\mu}_{it}={R}_{s, it}{\sum}_{l=1}^3{g}_{il}{\mu}_{it-l}$. That is ${\mu}_{i1}=2;{\mu}_{i2}=1;{\mu}_{i3}=6;{\mu}_{it}={R}_{s, it}{\sum}_{l=1}^t{g}_{il}{\mu}_{it-l},t>3$. The infectious time interval is also evaluated in the simulation study to examine the effect of different window sizes. We investigate the sensitivity of the window choice by assuming *L* = 2, 3 and 4 weeks in the simulation study because the infectious period of arthropod-borne diseases such as Dengue and Zika usually lasts for a couple of weeks [18]. The results displayed are from posterior sampling carried out on WinBUGS, user-friendly software, using MCMC with an initial burn-in period of 100,000 iterations to assess the convergence of MCMC chains.

**Fig. 3:**
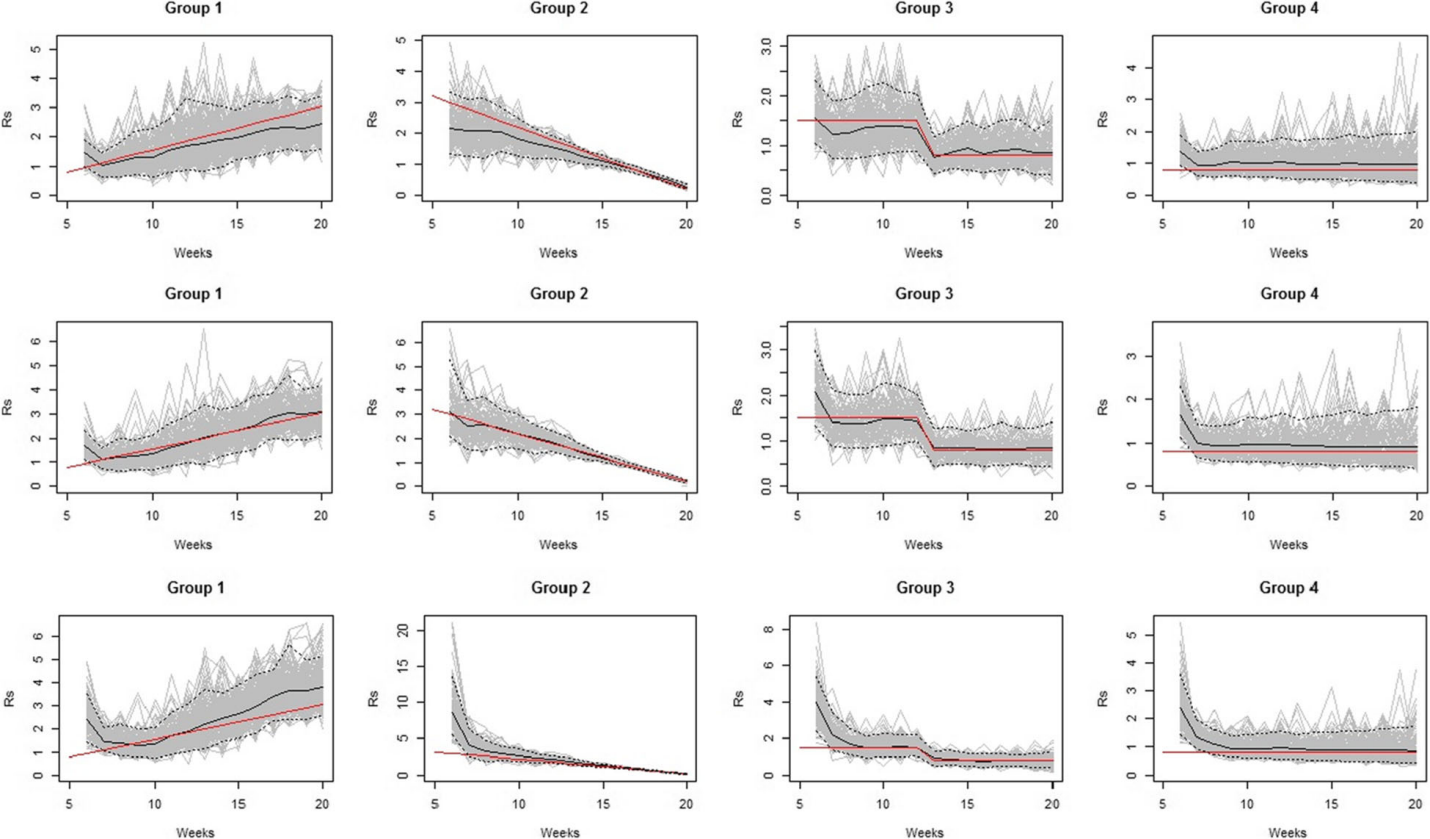
Plots of the posterior estimated *R*_*s*_ of all district groups with infectious periods of 2 (top), 3 (middle), and 4 (bottom) weeks from all simulated datasets. The black lines show the estimated mean with dash lines showing the 95% credible interval. The grey lines display posterior realizations and the red lines are the true *R*_*s*_ used for simulation

**Fig. 4:**
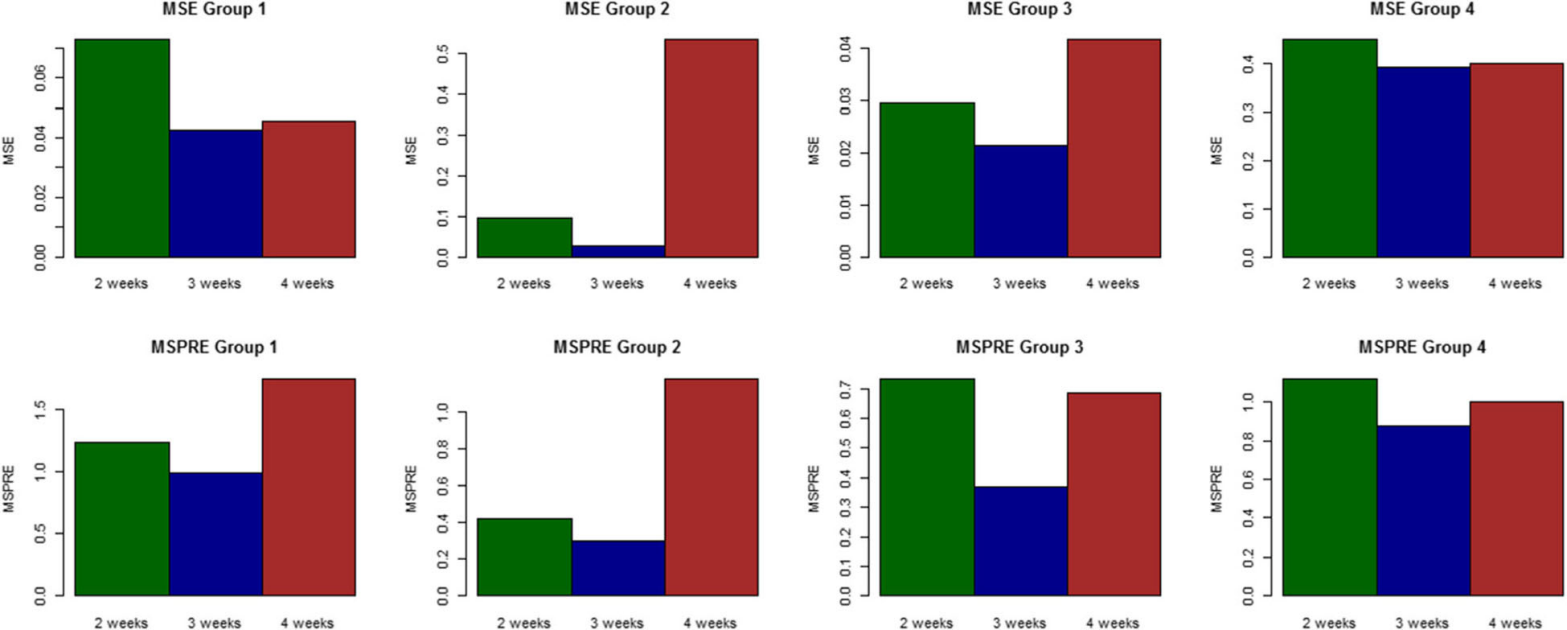
Bar plots of MSE and MSPRE of the estimated and predicted reproduction number with different infectious times of four district groups

The simulated and corresponding estimated *R*_*s*_ for each district group with different infectious times are shown in Fig. 3. Our methodology allows estimating a constant surveillance reproduction number used for simulation in scenario 4. The constant changes in *R*_*s*_ are detected in both increasing (scenario 1) and decreasing (scenario 2) force of infection. It also can effectively identify a jump in transmissibility (scenario 3). Figure 4 (top row) displays the mean squared error (MSE) of the estimated surveillance numbers in all scenarios and the correct infectious time, *L* = 3, yields the most precise estimate (the least MSE).

The estimate of the surveillance number also depends on the choice of the time window size *L*. However, it may not be feasible to know the true infection time in real situations. Then we examine a loss function metric which employs the predictive distribution to guide selecting the infectious profile. A commonly used loss function is the mean squared predictive error (MSPE) [19] comparing the observed data to the predicted values from a fitted model. However we are interested in the loss function in estimation of *R*_*s*_ . We then propose another predictive loss function, the mean squared predictive reproduction error (MSPRE), to evaluate the model predictive adequacy in terms of reproduction number defined as.

${MSPRE}_{it}={\sum}_{n=1}^N{\left({R}_{s, itn}^y-{R}_{s, itn}^{y^{pred}}\right)}^2/N$, where ${R}_{s, itn}^y=\frac{y_{it}}{\sum_{l=1}^L{g}_{inl}{y}_{it-l}}$, ${R}_{s, itn}^{y^{pred}}=\frac{y_{int}^{pred}}{\sum_{l=1}^L{g}_{inl}{y}_{\mathit{\operatorname{int}}-l}^{pred}}$, *N* is the size of posterior sampler and ${y}_{itn}^{pred}$ is generated from its posterior predictive distribution at the *n th* posterior sampling after burn-in period. Figure 4 (bottom row) presents the MSPRE for district groups corresponding to different choices of infection time window. We can see the infectious time of 3 weeks has both the least MSE and MSPRE. Thus this metric can provide guidance on which the time windows to consider in practice. The use of MSPRE will also be demonstrated in a case study provided later. It should be noted that the time elapsed since infection, *l*, could vary by individual. However we model the aggregated count conditional on spatial units instead of at individual level. Then the infectious time would be averaged over an area. Given the sampling framework it is reasonable to assume a constant infectious time for the population. Nonetheless it is also possible that the infectious time has a spatiotemporal distribution over the study area which is perhaps dependent on environmental or demographic variables. Then the covariates should be included in the model when available as well.

As presented we have developed a robust methodology to estimate disease transmissibility varying across locations and time periods. Our method allows for computational flexibility not affected by conventional restrictions which generally are difficult to apply in real situations. However due to very limited knowledge when new emerging infection initially occurs, it is extremely challenging for policy makers to make decision based upon enormous uncertainty. Therefore it is essential to consider the analysis integrating relevant information streams in order to develop the best disease-management plans possible. Hence in the next section we extend the univariate framework to allow for incorporating knowledge from related diseases.

### Multivariate surveillance reproduction number

Limitations in availability of disease information constrain public health efforts to prevent and control outbreaks. Thus utilizing knowledge, we have from other sources such as related diseases can principally help improving the surveillance system. Dengue is one of the major arthropod-borne diseases in tropical and sub-tropical regions. The virus belongs to the genus *Flavivirus* and is primarily transmitted by *Aedes* mosquitoes as well as Zika. Similarity in virological characteristics and identification as etiologic agents of the similar illness and their co-infection suggest that these 2 *Aedes* mosquito-transmitted viruses can be circulating in the same area confirming the underlying potential for Zika to have a similar spreading pattern to Dengue [20, 21]. Therefore, in this section we develop a multivariate transmissibility measure allowing for transferring of spatiotemporal knowledge of these two flaviviruses in order to maximize the surveillance ability which was not considered in previous literature.

In the multi-disease surveillance setting, spatial data on multiple diseases are observed at each time period. We assume that *y*_*it*_ and ${y}_{it}^s$ are the number of new Zika and Dengue (with superscript) cases which are Poisson distributed with means *μ*_*it*_ and ${\mu}_{it}^s$ for each area *i* and time *t*. Using a logarithmic link function, *α* and *α*^*s*^ are the overall intercepts, and ***X***_*it*_***β***_*it*_ and ${\boldsymbol{X}}_{it}^s{\boldsymbol{\beta}}_{it}^s$ are the covariate predictors for both diseases. In general, once multiple diseases are introduced into an analysis there is a need to consider relations between the diseases. This can be achieved in various ways. A basic approach to this is to consider cross-correlation between the diseases [22, 23]. There is a numerous literature in the specification of cross-disease modeling using Gaussian process [24]. The multivariate conditional autoregressive (MCAR) model [25] and the shared component model [26] are the two primary approaches to model disease risk correlations across both spatial units and diseases. Here we use an extended version of the convolution model [27] to incorporate diseases’ correlation using multivariate spatially-correlated, *u*_*i*_ and ${u}_i^s$, and non-correlated, *v*_*i*_ and ${v}_i^s$, random effects to account for unobserved confounders and spatial variation. To capture the temporal trend a multivariate autoregressive prior distribution, which allows for sharing the temporal information between the diseases, is assumed for the temporal random effects, *λ*_*t*_ and ${\lambda}_t^s$. In addition, there is a space-time interaction term for each disease, *δ*_*it*_ and ${\delta}_{it}^s$, which are assumed to have a Gaussian prior distribution. Finally infectivity profiles *g*_*il*_ and ${g}_{il}^s$ are jointly approximated by a standardized multivariate Log-Normal distribution. A multivariate extension of the reproduction number, *R*_*ms*, *it*_, incorporating information from both diseases can be defined as ${R}_{ms, it}=\frac{\mu_{it}}{\int_0^{\infty }{g}_{it}(l){\mu}_{it-l} dl}\approx \frac{\mu_{it}}{\sum_{l=1}^L{g}_{il}{\mu}_{it-l}}$ and ${R}_{ms, it}^s=\frac{\mu_{it}^s}{\int_0^{\infty }{g}_{it}^s(l){\mu}_{it-l}^s dl}\approx \frac{\mu_{it}^s}{\sum_{l=1}^L{g}_{il}^s{\mu}_{it-l}^s}$. Then full specification of the joint modeling of Zika and Dengue is following:

8 $$ {\displaystyle \begin{array}{l}{y}_{it}\sim Poisson\left({\mu}_{it}\right);{y}_{it}^s\sim Poisson\left({\mu}_{it}^s\right)\\ {}\log \left({\mu}_{it}\right)=\alpha +{X}_{it}{\beta}_{it}+{u}_i+{v}_i+{\lambda}_t+{\delta}_{it}\\ {}\log \left({u}_{it}^s\right)={\alpha}^s+{X}_{it}^s{\beta}_{it}^s+{u}_i^s+{v}_i^s+{\lambda}_t^s+{\delta}_{it}^s\\ {}\alpha \sim N\left(0,{\tau}_{\alpha}^{-1}\right);{\alpha}^s\sim N\left(0,{\tau}_{\alpha^s}^{-1}\right)\\ {}{\beta}_{it}\sim N\left(0,{\tau}_{\beta}^{-1}\right);{\beta}_{it}^s\sim N\left(0,{\tau}_{\beta^s}^{-1}\right)\\ {}\left[\begin{array}{c}{u}_i\\ {}{u}_i^s\end{array}\right]\sim MCAR\left({\sum}_u^{-1}\right);\left[\begin{array}{c}{v}_i\\ {}{v}_i^s\end{array}\right]\sim MVN\left(\begin{array}{c}0\\ {}0\end{array},{\sum}_v^{-1}\right)\\ {}\left[\begin{array}{c}{\lambda}_t\\ {}{\lambda}_t^s\end{array}\right]\sim MVN\left(\begin{array}{c}{\lambda}_{t-1}\\ {}{\lambda}_{t-1}^s\end{array},{\sum}_{\lambda}^{-1}\right)\end{array}}\kern0.5em {\displaystyle \begin{array}{l}{\delta}_{it}\sim N\left(0,{\tau}_{\delta}^{-1}\right);{\delta}_{it}^s\sim N\left(0,{\tau}_{\delta^s}^{-1}\right)\\ {}{R}_{ms, it}=\frac{\mu_{it}}{\sum_{l=1}^L{g}_{il}{\mu}_{it-l}};{R}_{ms, it}^s=\frac{\mu_{it}^s}{\sum_{l=1}^L{g}_{il}^s{\mu}_{it-l}^s}\\ {}{g}_{il}=\frac{\exp \left({w}_{il}\right)}{\sum_{l=1}^L\exp \left({w}_{il}\right)}{g}_{il}^s=\frac{\exp \left({w}_{il}^s\right)}{\sum_{l=1}^L\exp \left({w}_{il}^s\right)}\\ {}\left[\begin{array}{c}{w}_{il}\\ {}{w}_{il}^s\end{array}\right]\sim MVN\left(\begin{array}{c}0\\ {}0\end{array},{\Sigma}_w^{-1}\right)\\ {}{\tau}_{\ast}^{-1/2}\sim Unif\left(0,10\right).\end{array}} $$

## Results

### Application to dengue and Zika virus surveillance activities in Thailand

Dengue is endemic in Thailand with peak transmission rates occur in the rainy season, between June and September, all across the country, but particularly in northeastern Thailand. Zika was first reported in Thailand in 2012, and the Bangkok Metropolitan Authority has been conducting regular screen tests on its residents since then. To demonstrate performance of the proposed integrative method we apply the multivariate surveillance number, *R*_*ms*_, to the Dengue and Zika prevalence in Thailand. The cases of both diseases were from the province of Chanthaburi consisting of 10 health districts during July 10th until August 27th 2016, total of 7 weeks. The information of case patients was reported by the public hospitals to surveillance center. Note that the dengue patients included in this analysis were both who diagnosed with Dengue fever (DF) and Dengue hemorrhagic fever (DHF). The results displayed are based on the approximation of the surveillance number developed using prevalence in (6) and posterior sampling carried out using WinBUGS software an initial burn-in period of 100,000 iterations to assess the convergence of MCMC chains.

The estimates of the surveillance numbers are expected to depend on the choice of the size of infectious time *l*. The *Aedes aegypti* mosquito is the primary vector of Dengue. The virus is transmitted to humans through the bites of infected female mosquitoes. After virus incubation for 4–10 days, an infected mosquito is capable of transmitting the virus for the rest of its life. Infected symptomatic or asymptomatic humans are the main carriers and multipliers of the virus, serving as a source of the virus for uninfected mosquitoes. Patients who are already infected with the dengue virus can transmit the infection (for 4–5 days; maximum 12 days) via *Aedes* mosquitoes after their first symptoms appear. Zika is usually milder with symptoms lasting for several days to a week. People usually don’t get sick enough to go to the hospital, and they very rarely die of Zika [18].

MSPRE is applied to guide on the choice of infectious time for the model. Table 1 displays the values of MSPRE of both diseases fitted with different sizes of the infectious times. The window size of 2 weeks fitted with the univariate model yields the least MSPRE for Zika and Dengue. Though based on the clinical manifestation point of view a window size less than 2 weeks may be possible for Zika, the result suggested by MSPRE is sensible combining with knowledge from epidemiological perspective that incubation period and virus lifespan in a mosquito can prolong the infectious period. Nonetheless we have only weekly data and would recommend using a finer temporal scale if appropriate when such data are available. To evaluate the performance, we compare the univariate and multivariate models weekly so that both models would have the same set of data. Based on the guidance from MSPRE and information discussed earlier, we thus choose the size of infectious time to be 2 weeks for both diseases in the analysis. Because the infectious times are assumed to be 2 weeks, for simplicity, we assume the weights of serial intervals to have a Beta (1,1) prior distribution instead of standardized log-normal distribution. All covariance matrices are assumed to be a fixed matrix of $$ \left[\begin{array}{cc}100& 0\\ {}0& 100\end{array}\right] $$ which however could also be modeled with a Wishart distribution as well.

**Table 1:** MSPRE of Dengue and Zika fitted with the univariate model for different time windows

	Infectious time (weeks)		
	2	3	4
Dengue	0.6814	0.6992	0.7018
Zika	0.2693	0.2998	0.3110

Table 2 presents DIC values obtained from the analysis with both the univariate and multivariate models during weeks 4–7. The DIC of the multivariate model is less than the ones from each disease fitted separately by the univariate model across all time periods. Moreover, pD, which can be seen as model complexity, is also much smaller in the case of multivariate model. This suggests that pooling information from both diseases in the analysis essentially decreases variability in model fitting and provides a much better description of the spreading pattern of Zika and Dengue.

**Table 2:** DIC (pD) values for Dengue and Zika fitted with univariate and multivariate models during weeks 4–7

		Week 4	Week 5	Week 6	Week 7
Univariate	Dengue	204.4 (78.25)	217.01 (72.08)	232.45 (73.82)	230.09 (62.63)
	Zika	103.1 (37.62)	247.3 (108.44)	519.8 (241.21)	1505.6 (731.1)
Multivariate	Dengue	85.35 (15.91)	119.65 (19.78)	134.98 (21.79)	155.67 (23.52)
	Zika	52.16 (7.39)	62.64 (10.1)	79.37 (13.42)	86.02 (14.47)

**Fig. 5:**
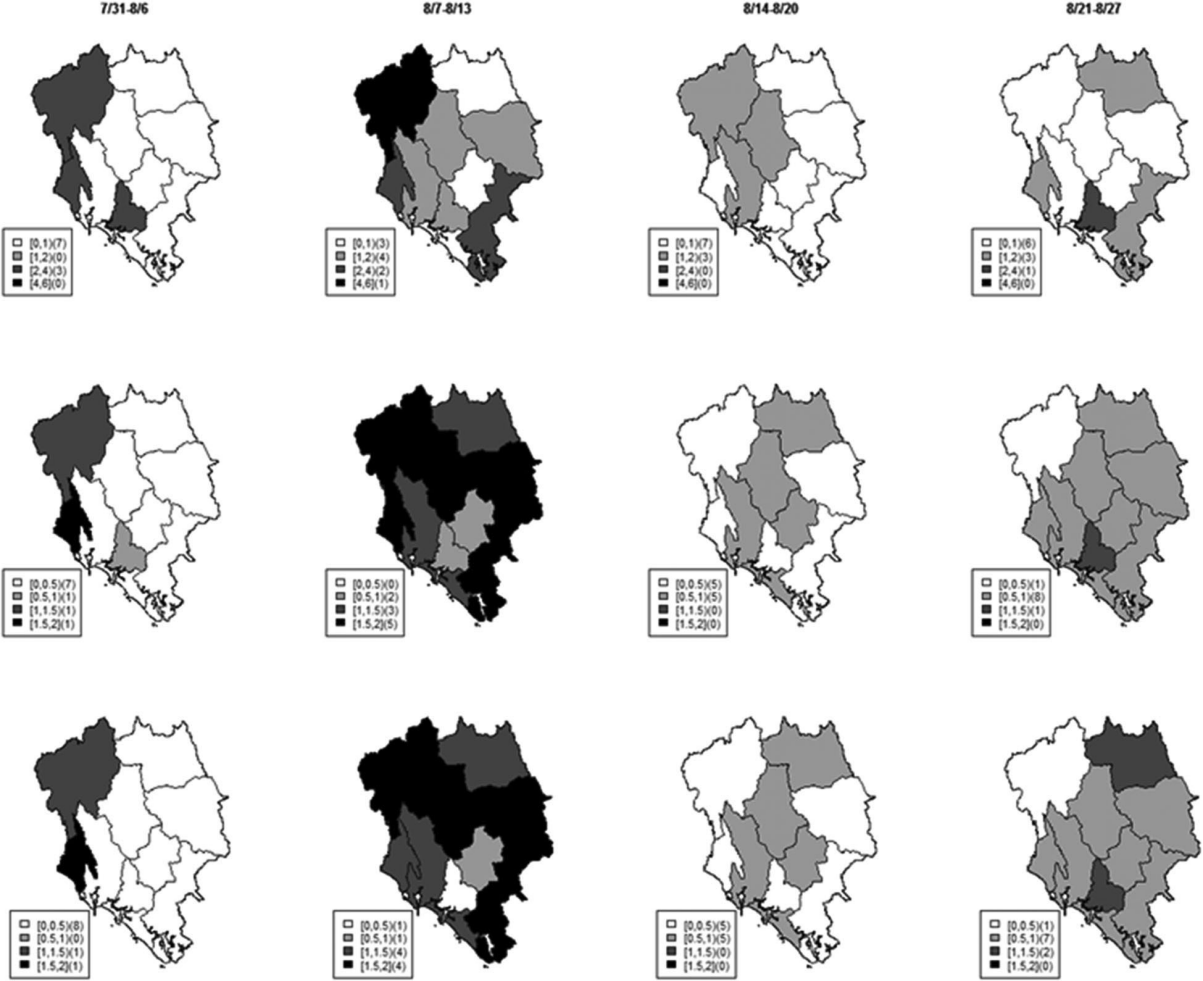
Maps of weekly Dengue incidence (top), *R*_*s*_ (middle), and *R*_*ms*_ (bottom) in Chantaburi during July 31st – August 27th 2016

**Fig. 6:**
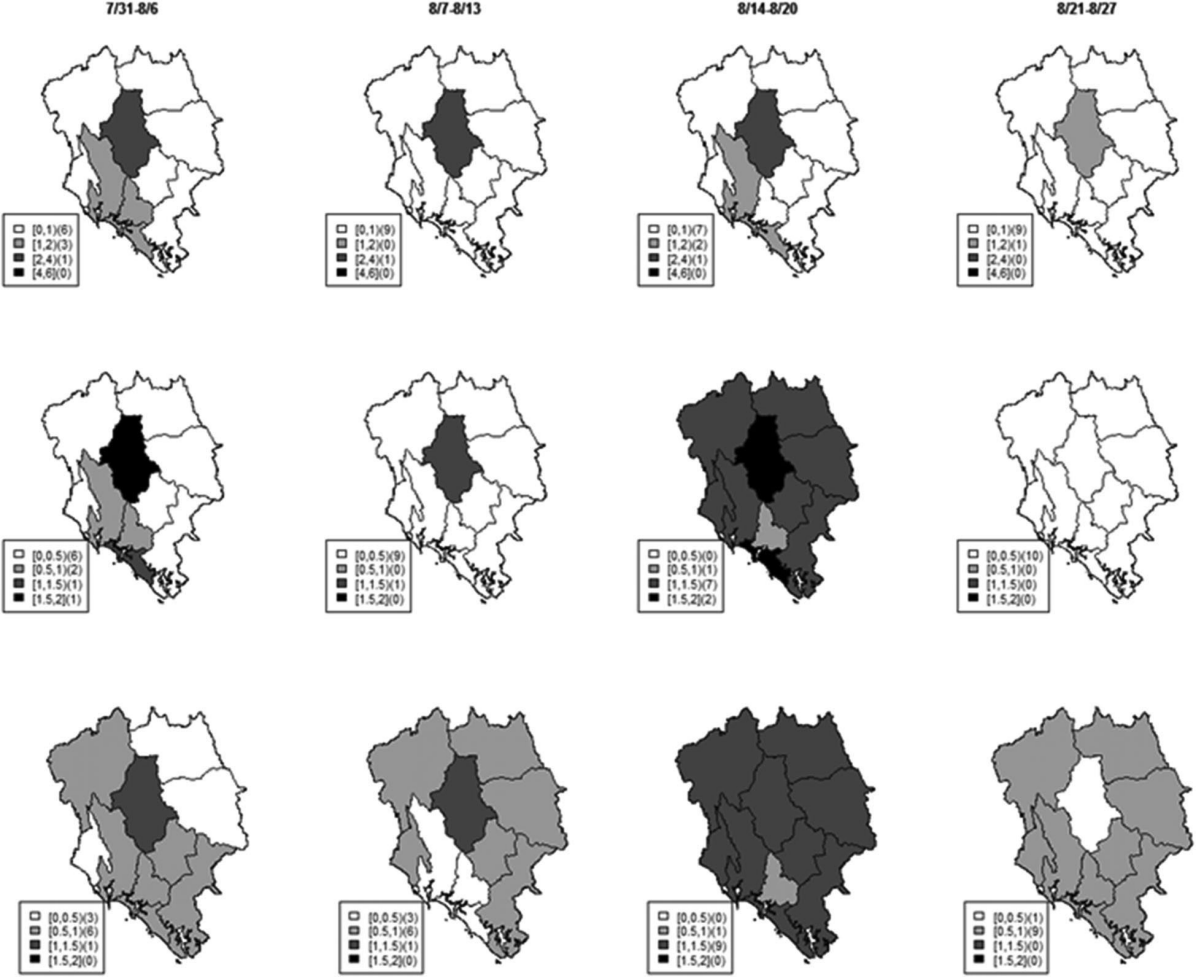
Maps of weekly Zika incidence (top), *R*_*s*_ (middle), and *R*_*ms*_ (bottom) in Chantaburi during July 31st – August 27th 2016

The estimated *R*_*s*_ and *R*_*ms*_ describe the pattern of Dengue transmission similarly. However *R*_*ms*_ (bottom row, the second column in Fig. 5) of Dengue, which also infuses information of Zika pattern in the integrative platform, provides a smaller transmission rate than *R*_*s*_ (middle row, the second column in Fig. 5) at a district in the south during the week of August 7th – August 13th. This is because during the same week the number of Zika incidence at that district (the first row, second column in Fig. 6) was decreasing from the previous week (the first row, first column in Fig. 6). On the other hand, during the week of August 7th – August 13th Dengue incidence was increasing from the previous week. Hence the *R*_*ms*_ of Zika estimated from the multivariate model suggests a higher estimate of the disease strength than from the univariate model. These results demonstrate that the proposed integrated model allows for transferring transmission knowledge between the related diseases to optimize surveillance ability.

## Discussion

A new emerging disease can occur in one place and have the potential to spread globally. This is an important challenge in terms of emergency preparation requiring the operation of surveillance systems. A traditional concept to study the dynamic of infectious diseases is the basic reproduction number. However, the intuitive appeal of its theoretical interpretation can outlast the appropriateness of situations if applied incautiously. So it is remarkably crucial to be aware of their caveats when adopting that measure. Otherwise, it could mislead to the inappropriate backbone of disease-management policy. Alternatively, we present a robust and flexible methodology for estimating spatiotemporally varying reproduction numbers. Withstanding the issues of context-specific assumptions, our method provides more practical advantages and can be used to simultaneously estimate disease transmissibility for each location and time within a user-friendly environment for real-time assessment of new emerging diseases.

To evaluate our method, we simulate data in several situations with different magnitudes of transmission and sizes of infectious period. The simulation results suggest that the proposed method allows robust estimation of the surveillance reproduction number used for simulation across simulated scenarios. Though from the simulation study the method would not suffer much from the infection window size, MSPRE may be helpful in providing guidance on the choice of infectious period in practice. Due to limited information when new emerging infection newly occurs, the univariate framework is extended to allow for incorporating knowledge from related diseases in order to maximize the surveillance capability. A case study is provided of an integrative application of Dengue and Zika surveillance in Thailand.

A significant portion of arbovirus incidence (eg. ZIKA) is underestimated due to asymptomatic infection without presenting any clinical symptoms [28]. Nevertheless, the contribution of asymptomatic reservoirs to the overall disease burden has not been well quantified, which introduces considerable uncertainty into modeling studies of disease transmission dynamics and control strategies. Policy and practice on case detection and reporting of Dengue and Zika is a critical factor due to the nature of diseases that have high proportion of asymptomatic infection. Therefore novel surveillance tools, such as integrated surveillance, should be developed and applied to improve estimates of disease incidence especially asymptomatic infections such as Dengue and Zika.

The significance of our development lies in the advantages of multivariate surveillance in the ability to borrow strength across diseases and to allow conditioning of one on others. When applying the multivariate framework, the relevant diseases should epidemiologically and clinically sound. Studies indicate the underlying possibility for Zika to have a similar spreading pattern to Dengue [2, 20]. We hence extend our method to allow for integrating related diseases’ information and also demonstrate its performance in the example of Dengue and Zika surveillance in Thailand. The data example shows that combining both diseases in an integrated analysis essentially decreases variability of model fitting. The result suggests that the proposed integrative platform which allows for transferring transmission knowledge between the related diseases sharing similar etiology not only can enhance the estimation of transmissibility but also helps explaining the spreading pattern of Zika and Dengue much better. This is a significant importance of the proposed multi-disease measure in improving surveillance ability.

Though the proposed method demonstrates robust performance, it should be noted that those data present a lot of both clinical and epidemiological complexity. In this work we prevalence information is assumed for the model due to difficulties of disease investigation which implies that the ratio of incidence and prevalence is nearly constant over time. This is a limitation of our development. This assumption may not be appropriate for chronic diseases but rather suitable for infections with relatively short duration. There is a further need for studies of virus circulation persistence and the ecological factors including characterizing immunological cross-reacting which could shorten or prolong the epidemic [29]. Across both clinical and ecological studies, it is also important to evaluate the effect of host, viral, and vector relationships for fuller understanding of the disease mechanism [18]. However, the proposed methodology can be served as a flexible platform to incorporate those potential epidemiologic and ecologic determinants that drive the disease risk as they are available.

## Conclusions

New emerging diseases are public health crises in which policy makers have had to make decisions in the presence of massive uncertainty. As presented the proposed methodology is robust in several simulated scenarios of transmission force with computing flexibility and practical benefits. Thus this development is ideally suitable for surveillance applications of new emerging diseases such as Zika. To further prevent and control new emerging infection, we must have a fuller understand the modes of transmission which are currently lacking. In such context, it is natural to look for strategies that mirror those applied for relevant diseases. By transferring information from diseases sharing the similar etiology such as Dengue, our multivariate framework can successfully integrate knowledge and hence improve the surveillance system effectively. Therefore, in current situations whereas there are threats from new infection, a robust and flexible platform is thus essentially needed to be readily prepared in order to rapidly gain an understanding of the new disease transmission mechanism to counter the local and global health concerns.

## Data Availability

The data that support the findings of this study were obtained from the Thai Bureau of Epidemiology, but restrictions apply to the availability of these data, which were used with permission for the current study, and are therefore not publicly available. However, data may be available from the authors upon reasonable request and with permission of the Thai Bureau of Epidemiology.

## Abbreviations

DF: Dengue fever
DHF: Dengue hemorrhagic fever
DIC: Deviance Information Criterion
ICAR: Intrinsic Conditional Autoregressive model
MCAR: Multivariate Conditional Autoregressive model
MCMC: *Markov chain Monte Carlo*
MSE: Mean Squared Error
MSPE: Mean Squared Predictive Error
MVN: Multivariate Normal distribution
*R*_*0*_: Basic reproduction number
*R*_*ms*_: Multivariate surveillance reproduction number
*R*_*s*_: Surveillance reproduction number
WHO: World Health Organization
